# Report of a case of cavernous haemangioma of the cavernous sinus

**DOI:** 10.1259/bjrcr.20190031

**Published:** 2019-11-15

**Authors:** Dalia Ibrahim, Ahmed El Fiki, Mohamed Hafez, Sahar Saleem

**Affiliations:** 1 Department of Radiology, Lecturer of Radiodiagnosis, Kasr Al Ainy Hospital, Egypt; 2 Department of Neurosurgery, Assistant professor of Neurosurgery, Kasr Al Ainy Hospital, Egypt; 3 Professor and head of Neurosurgery department, Kasr Al Ainy Hospital, Egypt; 4 Department of Radiology, Professor of Radiodiagnosis, Kasr Al Ainy Hospital, Egypt

## Abstract

Cavernous haemangioma of the cavernous sinus is a rare vascular malformation. It's often confused with other parasellar masses. Here, we report a case of a female with a left parasellar mass which was misdiagnosed as schwannoma *vs* meningioma using CT and MRI. The patient was operated via the pterional approach but resection had been halted due to severe haemorrhage and only tumour biopsy could be obtained. The diagnosis of cavernous sinus haemangioma was established by histopathology and confirmed by subsequent digital subtraction angiography. The patient refused second surgery or adjuvant radiosurgery and the treatment strategy was observation and follow-up. Retrospectively, we included the key radiographic features of cavernous sinus haemangioma which would facilitate pre-operative diagnosis and avoid unforeseen operative complications. Diagnostic radiographic features include a well-defined mass in the cavernous sinus which shows isodense to slightly hyperdense attenuation on non-contrast CT scan with possible adjacent pressure bone remodelling. On MRI, it shows remarkable high *T*_2_ signal; intense homogenous enhancement or characteristic progressive contrast enhancement on sequential enhanced images. On digital subtraction angiography, it may demonstrate a vascular blush.

## Case presentation

A 48-year-old female presented with a history of progressive headache and blurring of vision of 1 year duration. Her physical and neurological examinations were unremarkable. Routine laboratory investigations and pituitary hormonal profile were all normal.

### Investigations

**Figure 1.**
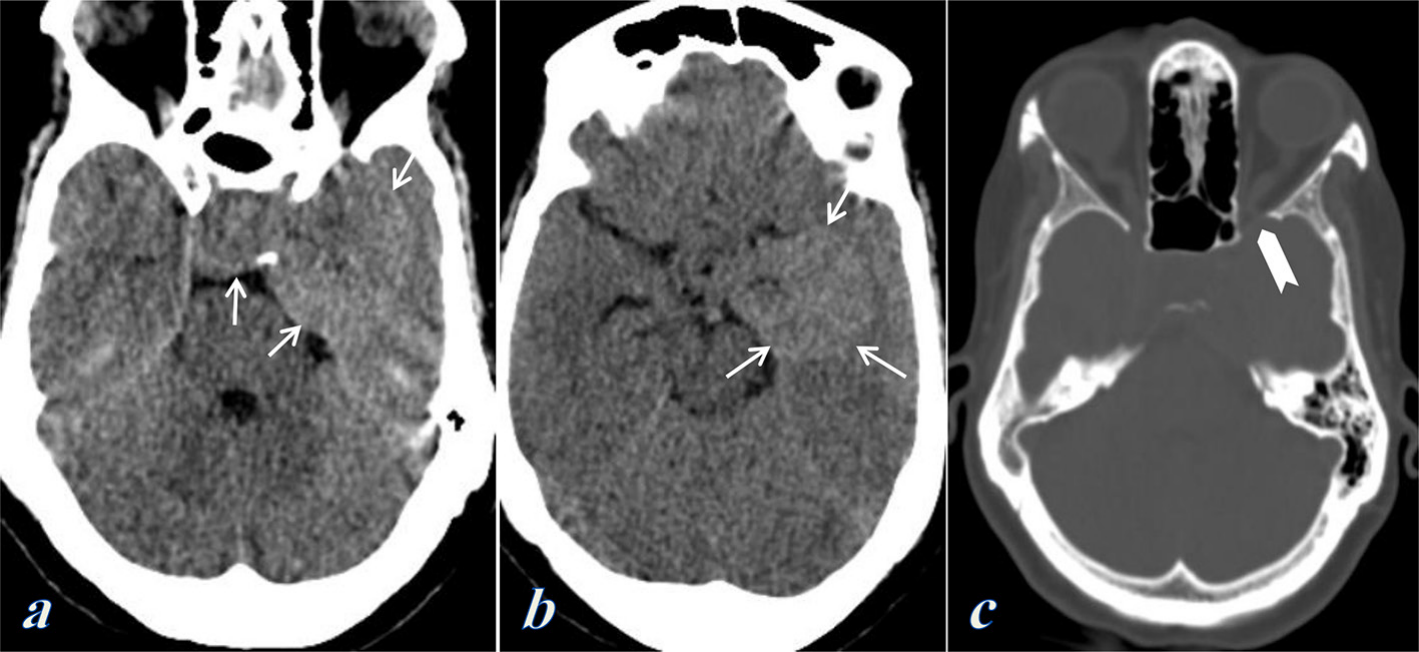
Non-contrast CT scan of the brain; (a, b) Axial soft tissue window showed a large extra axial sellar and left parasellar mass which is isodense/slightly hyperdense to the brain parenchyma (Arrows). It effaces the prepontine cistern, (c) Axial bone window showed bony scalloping and erosion of the medial part of the sphenoid wing (Chevron).

Non-contrast CT scan of the brain revealed a large extra axial mass at the sellar and left parasellar regions. It appears homogeneous and shows isodense to slightly hyperdense attenuation relative to the adjacent brain parenchyma. No internal calcifications or necrosis. The lesion effaces the prepontine cistern. Examination of the bone window showed smooth bone remodelling and erosion of the medial part of the sphenoid wing. No evidence of bone hyperostosis. (Figure 1)

**Figure 2.**
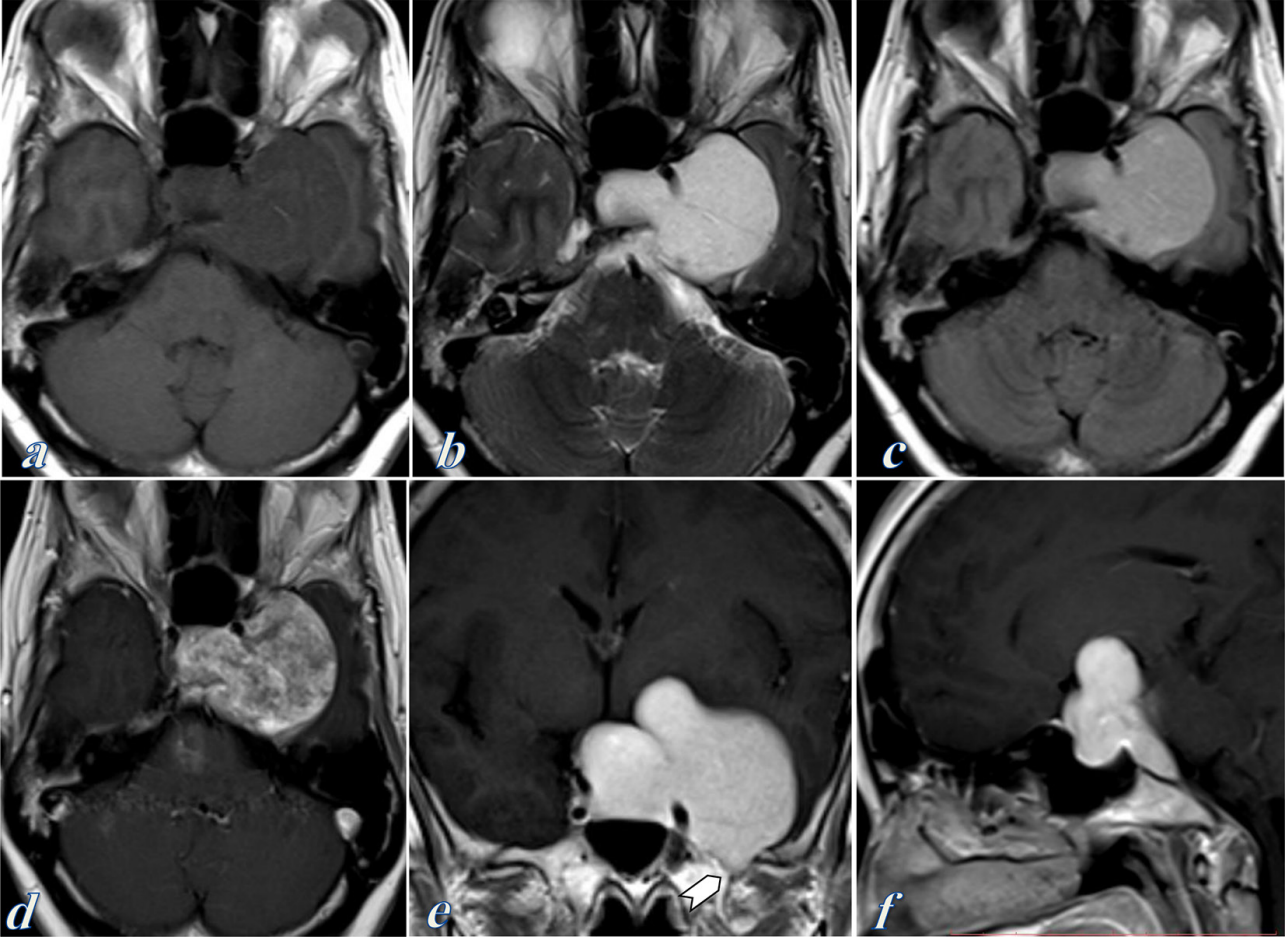
Contrast-enhanced MRI examination of the brain. (a) Pre-contrast axial *T*_1_ weighted image showed a large extra axial dumbbell sellar and left parasellar mass which elicits isointense to low signal intensity. (b, c) Axial *T*_2_ and FLAIR-weighted images showed remarkably high signal intensity of the mass. (d) Initial gadolinium-enhanced axial *T*_1_ weighted image showed heterogeneous enhancement of the mass. (e, f) Subsequent contrast-enhanced coronal and sagittal *T*_1_ weighted images showed intense homogeneous enhancement of the mass. The lesion causes enlargement of the left foramen ovale (Chevron in e). The cavernous internal carotid artery is mildly displaced laterally and is encased by the lesion without occlusion.

MRI following gadolinium contrast administration showed an extra axial well-demarcated dumbbell mass at the sellar and left parasellar regions. It measures 5.2 × 4.3 cm at its maximum transverse and anteroposterior diameters. The lesion displaces the temporal lobe laterally and extends medially into the sellar and suprasellar regions. The mass elicits low signal on *T*_1_ weighted images and remarkable high signal on *T*_2_ weighted images (Figure 2). Following contrast administration, it showed heterogeneous enhancement initially on the axial post-contrast sequence followed by homogeneous intense enhancement on the subsequently obtained coronal and sagittal sequences. No diffusion restriction on diffusion-weighted images (DWI). No blooming effect on gradient-echo (GRE) sequences. The cavernous internal carotid artery is encased by the lesion without occlusion.

### Differential diagnosis

Pre-operative neuroradiologic diagnosis after MRI was:

- Trigeminal schwannoma
- Meningioma
- Pituitary macroadenoma with parasellar extension

### Treatment and follow up

**Figure 3.**
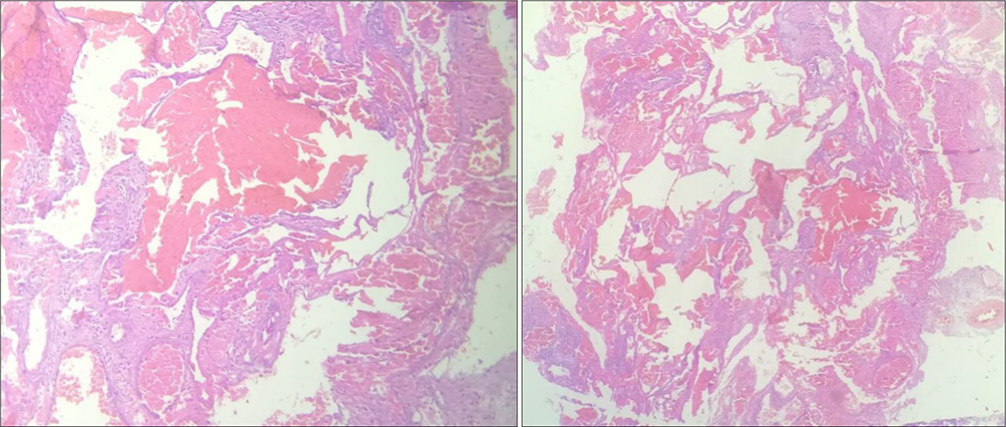
Photomicrographs showing dilated cavernous type vascular spaces lined by flat endothelial lining. Most of the vascular spaces were filled with blood cells and others were empty.

The patient went on surgery to have resection via transcranial pterional approach but this had been halted due to severe bleeding and only tumour biopsy could be obtained. Histopathological examination of the mass revealed soft tissue lesion formed of dilated (cavernous type) vascular spaces lined by flat endothelial lining. Some vascular spaces were empty but most spaces were filled with blood cells (Figure 3); a picture of cavernous haemangioma.

**Figure 4.**
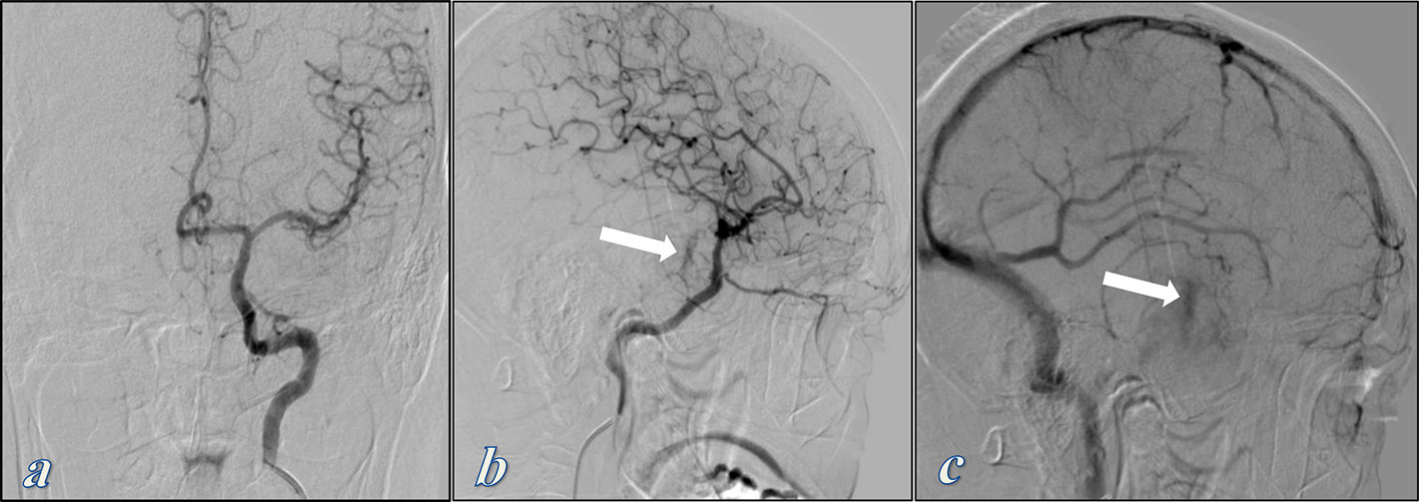
Cerebral angiography of the left internal and external carotid arteries. (a) Frontal projection shows only mass effect on the cavernous internal carotid artery which is mildly displaced laterally and on the middle cerebral artery which is mildly displaced superiorly. (b, c) Lateral projections at the late arterial and venous phases show tumor blush in the late arterial phase which becomes more evident in the venous phase (White arrows) . It is supplied by small meningeal branches of the internal carotid artery.

Following surgery, digital subtraction angiography was performed and showed a tumour blush at the lateral projection during the late arterial and venous phases supplied from small meningeal branches of the internal carotid artery (Figure 4). It also showed a mass effect on the internal carotid artery which is mildly displaced laterally and on the middle cerebral artery which is mildly displaced superiorly.

### Outcome

Post-operative neurological examination revealed paralysis of the left third cranial nerve with ptosis in the left eye and loss of light reflex; however, it gradually improved. The patient refused second surgery for total excision of the lesion or adjuvant radiosurgery and preferred the strategy of follow up and observation.

## Discussion

Cavernous haemangiomas, also known as cavernous angiomas or cavernomas, are defined as vascular malformations formed of abnormal dilated vascular spaces with no intervening neural tissue.^1,2^ Cavernous haemangioma is one of the four most common types of cerebral vascular malformations.^2^ They commonly occur intra axially within cerebral parenchyma^1,3^ ; however, they can also occur within extra axial spaces and when they occur extra axially they have the propensity to develop at the cavernous sinus.^4^ Extra axial haemangiomas have the same histologic features as intra axial lesions but have a different clinical picture, radiologic findings, and management.^3^

Cavernous sinus haemangioma has a predilection to middle-aged females^5^ as in our case. Cavernous sinus haemangioma has not been reported before in the Egyptian population; however, several cases were documented in Japan.^1,4,6,7^ The clinical presentation is insidious and patients usually suffer from headache and dysfunction of the cranial nerves passing through the cavernous sinus, presenting by ptosis, diplopia or facial neuralgia. Optic chiasm may be compressed in large masses and patients may thus present with decreased visual acuity.^5^

Cavernous sinus haemangiomas are divided pathologically into three types. Type A is a sponge-like lesion with intact pseudocapsule which consists of thin-walled vascular sinusoids with scanty intervening connective tissue; this type usually demonstrates homogeneous contrast enhancement on MRI. Type B is a mulberry-like lesion with absent or incomplete pseudocapsule which contains ample solid parenchyma with well-formed vasculature and connective tissue, and Type C which has both Type A and Type B compositions. Types B and C usually demonstrate heterogeneous contrast enhancement on contrast enhanced MRI studies.^7,8^

Radiological imaging is essential for the prediction of cavernous sinus haemangioma and differentiation of haemangioma from similar intracavernous sinus masses. CT, MRI and digital subtraction angiography are helpful for diagnosis of cavernous sinus haemangioma.^7,8^ Jinhu and colleagues retrospectively reported MRI features of 21 surgically confirmed cavernous haemangiomas of the cavernous sinus. Most of the cases presented with a well-demarcated dumbbell-shaped appearance at the parasellar region extending to the middle cranial fossa laterally and sellar regions medially. Similar to our case, the cavernous haemangioma usually encircles the cavernous segment of the internal carotid artery without occluding it.^7^ Non-contrast CT shows a well-demarcated lesion of similar density or slightly higher density to the brain parenchyma (in contrast to meningioma which usually appears denser). It also shows intense homogeneous post-contrast enhancement. Calcifications are not common in haemangiomas however adjacent bony remodeling and erosion may happen.^2^ MRI reveals mass which elicits isointense or hypointense signal on *T*_1_ weighted images and remarkably hyperintense (CSF like) signal on *T*_2_ weighted images with homogeneous post-contrast enhancement.^1^ Some studies observed heterogeneous enhancement on the first contrast-enhanced series and subsequent homogeneous contrast enhancement or progressive “filling in” enhancement on subsequent contrast-enhanced series especially in histopathological types B and C.^7^ Unlike intra-axial cavernous angiomas, cavernous sinus haemangioma does not demonstrate *T*_2_ hypointense margin or blooming effect on gradient-echo sequences secondary to scanty hemosiderin deposition.^9^ Angiographically, haemangiomas exhibit a high degree of vascularity and a vascular blush has been reported^5^ ; however, they may be angiographically subtle.^2^ Tc-99m tagged red blood cell (RBC) imaging was recently recommended for the diagnosis of cavernous sinus haemangioma in the setting of nondiagnostic surgical biopsy.^8,9^

The differential diagnosis includes similar cavernous sinus masses.^2^ Our case was similar to meningiomas as it elicited homogeneous signal on *T*_1_ and *T*_2_ weighted images and was similar to trigeminal schwannomas as it elicited high *T*_2_ signal and caused widening of the ipsilateral foramen ovale; however, unlike meningiomas and schwannomas; the mass elicited characteristic remarkably high (CSF like) signal on *T*_2_ weighted images. Cartilaginous masses such as chondrosarcomas or chordomas are also hard to differentiate from cavernous sinus haemangiomas because they elicit high signal on *T*_2_ weighted images and demonstrate intense contrast enhancement; however, they are usually associated with calcifications and adjacent bone destruction.^10^ A characteristic MR feature of the lesion in our case was its dynamic enhancement pattern. Following contrast administration, the lesion showed early heterogeneous contrast enhancement on the initial axial images that changed to homogeneous enhancement on the subsequent coronal and sagittal post-contrast images. This progression of contrast enhancement took only the acquisition time for obtaining the contrast enhanced *T*_1_ weighted sequences (about 2 min per sequence); this can be explained by the pathological nature of the lesion being composed of thick-walled vascular sinusoids which caused this short delay to achieve homogeneous contrast enhancement. This rapid increase in the degree of contrast enhancement is characteristic for cavernous sinus hemangioma as it is not seen in other lesions^7^ ; angioblastic meningiomas, neurilemomas, and chondrosarcomas may display heterogeneous enhancement however they usually lack progressive “filling in” enhancement pattern or delayed homogeneous enhancement on subsequent sequences.^7^

Treatment of choice is surgical excision.^11^ However, the reported perioperative mortality rate was high due to uncontrollable severe bleeding.^12^ Therefore the pre-operative diagnosis is important because it requires the surgeon to be ready to tackle such pathology with low surface bipolar coagulation with no attempt to debulk the lesion without complete coagulation and shrinkage. Thus, the surgeon mindset should be delivered to that pathology.^1,2^ Also, piecemeal removal can be performed after the main feeding arteries are interrupted.^4^ Stereotactic radiosurgery or radiation therapy has been proposed as primary therapy for patients with inoperable lesions, for those that undergo limited subtotal resection as adjuvant therapy or as a pre-treatment for direct surgical excision as it suppresses tumour growth and decreases vascularity of the mass.^13,14^

## Learning points

1. Cavernous sinus cavernous haemangioma is a rare vascular malformation and an uncommon cause of cavernous sinus masses.
2. Differentiation of cavernous sinus haemangioma from similar intracavernous sinus masses like meningiomas and schwannomas is often challenging.
3. Preoperative diagnosis is crucial as it requires a different surgical technique to avoid intraoperative profuse bleeding.
4. MRI, CT and angiography studies can help to predict the diagnosis and to differentiate cavernous sinus haemangioma from similar cavernous sinus masses.
5. Cavernous sinus haemangioma on non-contrast CT scan appears as a mass in the cavernous sinus which shows isodense to slightly hyperdense attenuation with intense homogeneous contrast enhancement and possible adjacent smooth bone remodeling and erosion.
6. Cavernous sinus haemangioma has characteristic MRI features of remarkably high *T*_2_ signal; intense homogenous enhancement or characteristic progressive contrast enhancement on sequential enhanced images.
7. Angiographically, cavernous haemangiomas may show vascular blush as they exhibit a high degree of vascularity but sometimes they might be angiographically subtle.
